# Regulation of murine copper homeostasis by members of the COMMD protein family

**DOI:** 10.1242/dmm.045963

**Published:** 2021-01-08

**Authors:** Amika Singla, Qing Chen, Kohei Suzuki, Jie Song, Alina Fedoseienko, Melinde Wijers, Adam Lopez, Daniel D. Billadeau, Bart van de Sluis, Ezra Burstein

**Affiliations:** 1 Department of Internal Medicine, University of Texas Southwestern Medical Center, Dallas, TX 75390, USA; 2 Department of General Surgery, Tongji Hospital, Tongji School of Medicine, Shanghai 200065, China; 3 Section of Molecular Genetics, Department of Pediatrics, University of Groningen, University Medical Center Groningen, Antonius Deusinglaan 1, 9713 AV Groningen, The Netherlands; 4 Division of Oncology Research, College of Medicine, Mayo Clinic, Rochester, MN 55905, USA; 5 Department of Molecular Biology, University of Texas Southwestern Medical Center, Dallas, TX 75390, USA

**Keywords:** ATP7A, ATP7B, COMMD proteins, Copper homeostasis, Copper transporters, Endosomal trafficking

## Abstract

Copper is an essential transition metal for all eukaryotes. In mammals, intestinal copper absorption is mediated by the ATP7A copper transporter, whereas copper excretion occurs predominantly through the biliary route and is mediated by the paralog ATP7B. Both transporters have been shown to be recycled actively between the endosomal network and the plasma membrane by a molecular machinery known as the COMMD/CCDC22/CCDC93 or CCC complex. In fact, mutations in *COMMD1* can lead to impaired biliary copper excretion and liver pathology in dogs and in mice with liver-specific *Commd1* deficiency, recapitulating aspects of this phenotype. Nonetheless, the role of the CCC complex in intestinal copper absorption *in vivo* has not been studied, and the potential redundancy of various COMMD family members has not been tested. In this study, we examined copper homeostasis in enterocyte-specific and hepatocyte-specific COMMD gene-deficient mice. We found that, in contrast to effects in cell lines in culture, COMMD protein deficiency induced minimal changes in ATP7A in enterocytes and did not lead to altered copper levels under low- or high-copper diets, suggesting that regulation of ATP7A in enterocytes is not of physiological consequence. By contrast, deficiency of any of three COMMD genes (*Commd1*, *Commd6* or *Commd9*) resulted in hepatic copper accumulation under high-copper diets. We found that each of these deficiencies caused destabilization of the entire CCC complex and suggest that this might explain their shared phenotype. Overall, we conclude that the CCC complex plays an important role in ATP7B endosomal recycling and function.

## Abstract (precis)

**Summary:** Examination of copper homeostasis in enterocyte-specific and hepatocyte-specific COMMD gene-deficient mice revealed that homologs of COMMD1, which has been linked previously by genetic studies to copper regulation, also regulate copper handling in mammals.

## INTRODUCTION

Copper is an essential trace element that is required for the activity of various conserved enzymes that catalyze oxygen-dependent reactions. However, owing to its oxidative activity, excess copper is toxic to biological systems and, as a result, intracellular levels of copper are tightly regulated (Rae et al., 1999). In this regard, movement of copper across membranes is regulated carefully by the P-type ATPase transporters, ATP7A and ATP7B (Puig et al., 2002; Wang et al., 2011). In low-copper conditions, both transporters reside predominantly in the trans-Golgi network (TGN) and move to peripheral vesicles upon copper accumulation (Holloway et al., 2013; Yi and Kaler, 2014). The transporters reach the plasma membrane from these peripheral vesicles and deliver copper to the extracellular space. ATP7A is expressed in most tissues, including the intestine, where it is required for intestinal copper absorption, whereas ATP7B is expressed predominantly in the liver, where it is required for biliary copper excretion, the physiological route of copper elimination.

Genetic mutations in these copper transporters result in metabolic disorders of copper handling, in both humans and animals (Shim and Harris, 2003; Wang et al., 2011). Mutations in *ATP7A* (Menkes disease, MIM 309400) result in copper deficiency owing to impaired intestinal copper intake; conversely, mutations in *ATP7B* (Wilson's disease, MIM 277900) result in hepatic copper accumulation owing to impaired excretion by the liver. Besides these rare genetic diseases, other forms of copper metabolic disorders have been recognized in mammals, including dogs (Vonk et al., 2008). One of the best-studied copper metabolic disorder in dogs is an autosomal recessive disorder in Bedlington terriers (Su et al., 1982; Twedt et al., 1979). This disease is characterized by massive accumulation of copper in the liver, leading to chronic hepatitis and liver cirrhosis. The disorder is attributable to a genomic deletion of exon 2 of the copper metabolism MURR1 domain-containing 1 (*COMMD1*) gene, implicating COMMD1 in copper handling and excretion (van de Sluis et al., 2002). Indeed, hepatocyte-specific *Commd1* deletion in mice leads to elevated hepatic copper levels when animals are fed diets with high copper content (Vonk et al., 2011).

COMMD1 has been shown to be associated physically with ATP7A and ATP7B (de Bie et al., 2007; Vonk et al., 2012) and can regulate the protein stability of these transporters (Miyayama et al., 2010; Vonk et al., 2012). However, the precise mechanism by which it regulates copper homeostasis was elucidated only in recent studies (Phillips-Krawczak et al., 2015). It was revealed that COMMD1 is an essential component of a large protein complex containing two homologous coiled-coil domain-containing proteins, CCDC22 and CCDC93. This complex, named the COMMD/CCDC22/CCDC93 or CCC complex, is required for trafficking of ATP7A from the TGN to peripheral vesicles and to the plasma membrane in high-copper conditions (Phillips-Krawczak et al., 2015). In agreement with this, other studies demonstrated that COMMD1 also controls ATP7B trafficking in hepatoma cell lines (Miyayama et al., 2010; Stewart et al., 2019). Interestingly, in the case of ATP7B, these peripheral vesicles that ultimately fuse with the plasma membrane are lysosomes, and this process has been termed lysosomal exocytosis (Polishchuk et al., 2014). The CCC complex localizes primarily to the endosomes, and biochemical and genetic evidence indicates that it acts in concert with the Wiskott Aldrich Syndrome protein and SCAR homolog (WASH) complex (Wijers et al., 2019). WASH, like other members of the Wiskott Aldrich Syndrome protein, promotes Arp2/3-generated branched F-actin deposition, and directs this event to the cytosolic side of endosomal membranes. The CCC complex exerts its effects on protein sorting by limiting the activity of the WASH complex through regulation of phosphoinositide composition in the early endosomal compartment (Singla et al., 2019).

COMMD1 was the first identified member of the COMMD protein family, which consists of ten highly conserved proteins present from protozoa to humans. All COMMD proteins are defined by the presence of a unique C-terminal homology region termed the COMM domain (Burstein et al., 2005), which mediates COMMD-COMMD protein interactions and their incorporation into the CCC complex (Phillips-Krawczak et al., 2015). COMMD1 has been the focus of most studies to date, and much less is known about the function of other members of the family. All models of COMMD gene deletion studied thus far in mice result in embryonic lethality (Li et al., 2015; van de Sluis et al., 2007). Detailed characterization of the embryonic phenotypes revealed non-overlapping developmental defects and highlighted unique functions for these factors, including a role for COMMD1 in hypoxia-inducible factor regulation (van de Sluis et al., 2010, 2007) and the identification that COMMD9 regulates developmental events through regulation of Notch trafficking (Li et al., 2015).

More recent studies revealed that hepatocyte-specific *Commd1* deletion results in decreased hepatic uptake of low-density lipoprotein (LDL) cholesterol owing to poor recycling of the LDL receptor, which is degraded in lysosomes rather than recycled back to the cell surface (Bartuzi et al., 2016). Interestingly, other models of hepatocyte-specific COMMD gene deletion also shared this phenotype (Fedoseienko et al., 2018); these studies, along with observations in cell line models (Li et al., 2015), revealed that loss of individual COMMD proteins can destabilize multiple components of the CCC complex and, in effect, reduce the activity of the entire complex at once. However, besides studies on COMMD1, there is no knowledge on the function of other COMMD proteins or the CCC complex in copper handling in animals, nor prior demonstration *in vivo* that COMMD proteins regulate ATP7B localization in hepatocytes *in vivo*. Thus, we set out to study the potential physiological role of COMMD proteins in regulating ATP7B-mediated copper homeostasis.

## RESULTS

### COMMD protein deficiency destabilizes the CCC complex in enterocytes and hepatocytes

**Fig. 1.**
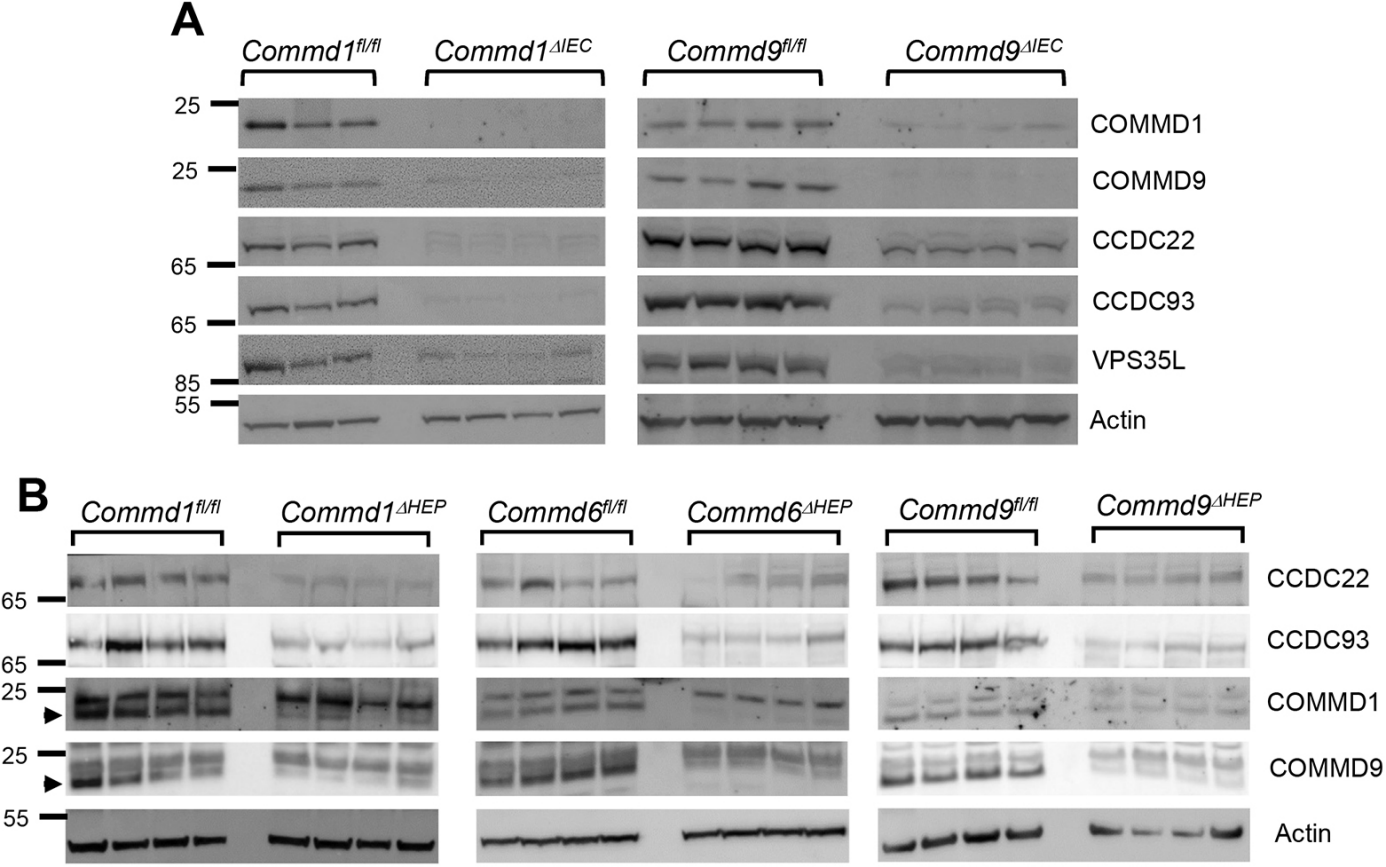
**COMMD protein deficiency causes the destabilization of the CCC complex in enterocytes and hepatocytes.** (A) Expression of CCC complex subunits was examined by immunoblotting in enterocytes isolated from intestinal-specific *Commd1* and *Commd9* knockout mice (*Commd1^ΔIEC^* and *Commd9^ΔIEC^*, respectively). Animals carrying the floxed alleles served as controls (*Commd1^fl/fl^* and *Commd9^fl/fl^*, respectively). β-Actin served as a loading control. (B) Same as in A, but using liver lysates from hepatocyte-specific COMMD-deficient mice (*Commd1^ΔHEP^*, *Commd6^ΔHEP^* and *Commd9^ΔHEP^*) and corresponding controls. Arrowheads indicate the band specific to the antibody.

Previously, using cell culture models, we have shown that the CCC complex regulates endosomal trafficking of the copper transporter, ATP7A (Phillips-Krawczak et al., 2015), the key copper transporter involved in intestinal absorption of copper (Wang et al., 2011). To understand the physiological role of COMMD proteins in regulating ATP7A *in vivo*, we deleted *Commd1* or *Commd9* from the intestinal epithelium (*Commd1^ΔIEC^* or *Commd9^ΔIEC^*) through crossing of mice with the corresponding floxed *Commd* alleles and the pan-intestinal Villin-Cre transgenic mouse. COMMD1 or COMMD9 deficiency in epithelial cells was validated using immunoblotting, as shown in Fig. 1A. Deficiency of COMMD1 or COMMD9 resulted in the depletion of core components of the CCC complex (CCDC22 and CCDC93), in addition to VPS35L (Fig. 1A), a subunit shared by the CCC and retriever complexes (Singla et al., 2019). Next, we compared these results with the expression of core components of CCC in liver lysates from mice that exhibit *Commd1*, *Commd6* or *Commd9* deletion specifically in hepatocytes [*Commd1^ΔHEP^*, *Commd6^ΔHEP^* or *Commd9^ΔHEP^* (Fedoseienko et al., 2018; Vonk et al., 2011)]. As shown in Fig. 1B, COMMD deletion in hepatocytes also resulted in the reduction of levels of the CCC complex, such as CCDC22, CCDC93 and other COMMD proteins. These results are consistent with prior cell culture models and studies in hepatocyte-specific knockouts (Fedoseienko et al., 2018).

### COMMD protein deficiency causes minimal alterations of enterocyte ATP7A *in vivo*

**Fig. 2.**
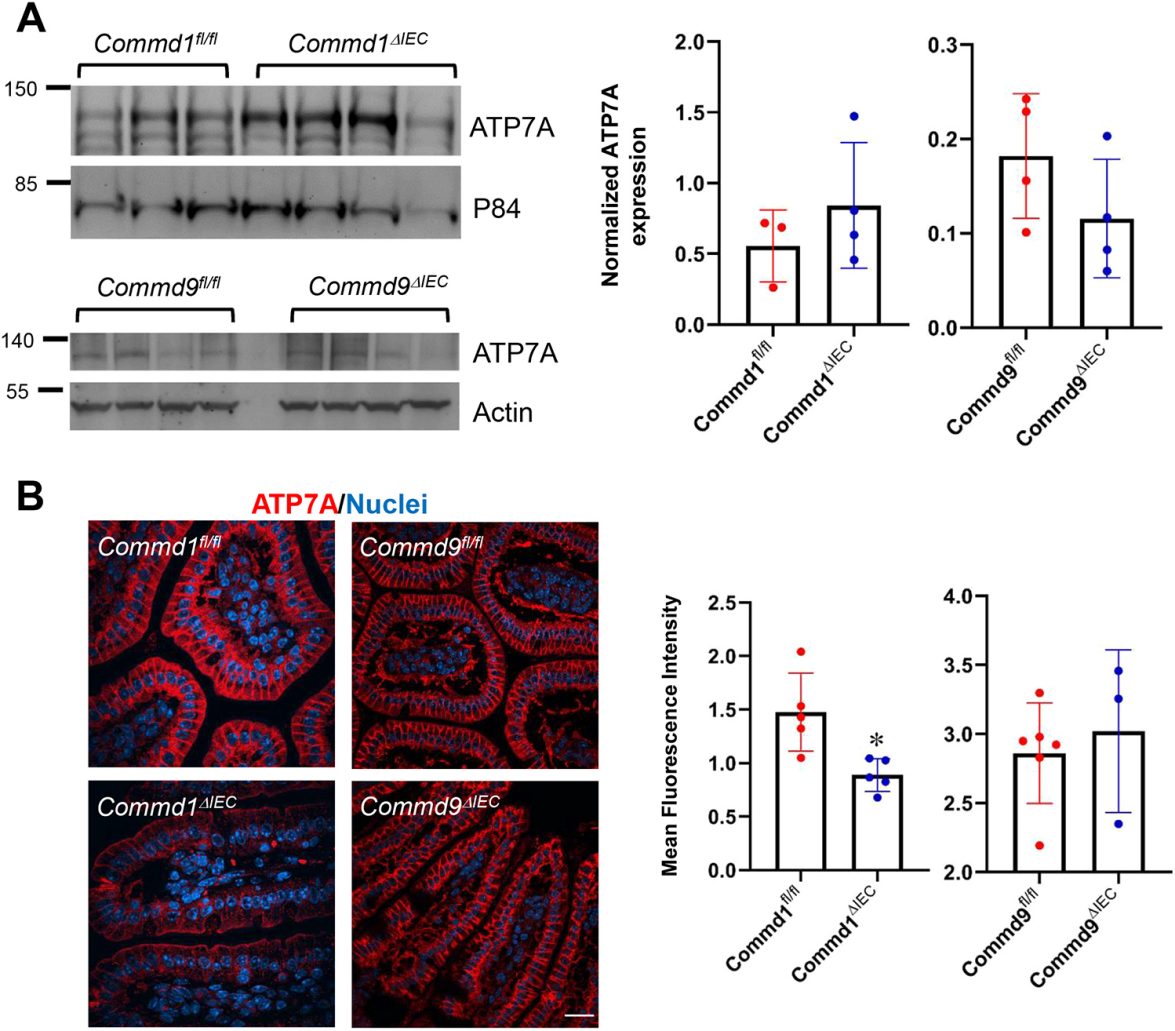
**ATP7A expression is diminished in *Commd1* but not *Commd9* intestinal knockout mice.** (A) Immunoblot analysis for ATP7A expression in *Commd1* and *Commd9* intestinal knockout mice (top). ATP7A quantification after normalization by the loading control (P84 or actin) (bottom). (B) Representative images of immunofluorescence staining for ATP7A (red) and nuclei (Hoechst, blue) in intestinal tissues of *Commd1^ΔIEC^* (*n*=5) or *Commd9^ΔIEC^* (*n*=3) mice (bottom row) or corresponding control floxed animals, *Commd1^fl/fl^* (*n*=4) and *Commd9^fl/fl^* (*n*=6) (top row). Scale bar: 25 µm. Bar graphs represent quantification of ATP7A signal intensity in immunofluorescence staining of intestinal tissues, expressed as the fold change over the nuclear staining. Results for individual mice are plotted along with the mean and s.e.m. for each group; *P<0.05 (unpaired two-tailed Student's *t*-test).

Next, we investigated the effect of COMMD protein deficiency on ATP7A expression and localization in enterocytes. Prior studies indicated that proteins that are not properly recycled from endosomes to the plasma membrane, such as integrins and Notch2, can undergo missorting to lysosomes and undergo protein degradation (Li et al., 2015; McNally et al., 2017). Immunoblot analysis demonstrated that there was no change in ATP7A protein levels in enterocyte lysates from *Commd1^ΔIEC^* or *Commd9^ΔIEC^* mice (Fig. 2A). Furthermore, we examined the localization of ATP7A in the intestinal epithelium by immunofluorescence staining. ATP7A localization was unchanged (Fig. 2B), but, in contrast to the immunoblotting data, there was a small reduction in ATP7A protein expression in *Commd1^ΔIEC^* but not *Commd9^ΔIEC^* mice, when compared with corresponding controls. Thus, despite global effects on CCC complex abundance caused by deficiency of either COMMD1 or COMMD9, there were only minor effects on enterocyte ATP7A levels observed in COMMD1-deficient enterocytes.

### COMMD or CCC deficiency alters hepatocyte ATP7B subcellular localization

To understand the role of COMMD proteins in regulating hepatic ATP7B, we first investigated the subcellular distribution of ATP7B in CCC-deficient Huh-7 cells. We examined this by comparing the localization of ATP7B in low- and high-copper conditions in parental Huh-7 cells and CCDC93-deficient Huh-7 cells. Parental Huh-7 cells demonstrated a significant redistribution of ATP7B in response to copper availability (Fig. S1): ATP7B localized to the TGN in low-copper conditions and moved to cytosolic vesicles in high-copper conditions. By contrast, CCDC93-deficient cells lost the normal redistribution pattern upon changes in copper concentrations. These cells displayed ATP7B in cytosolic vesicles in high- or low-copper conditions and lacked the TGN localization in low-copper concentrations. This phenotype is highly analogous to the impaired ATP7A trafficking observed in fibroblasts carrying a hypomorphic mutation in *CCDC22* or after silencing of COMMD1 (Phillips-Krawczak et al., 2015) and indicates an important role of the CCC complex in regulating ATP7B trafficking.

**Fig. 3.**
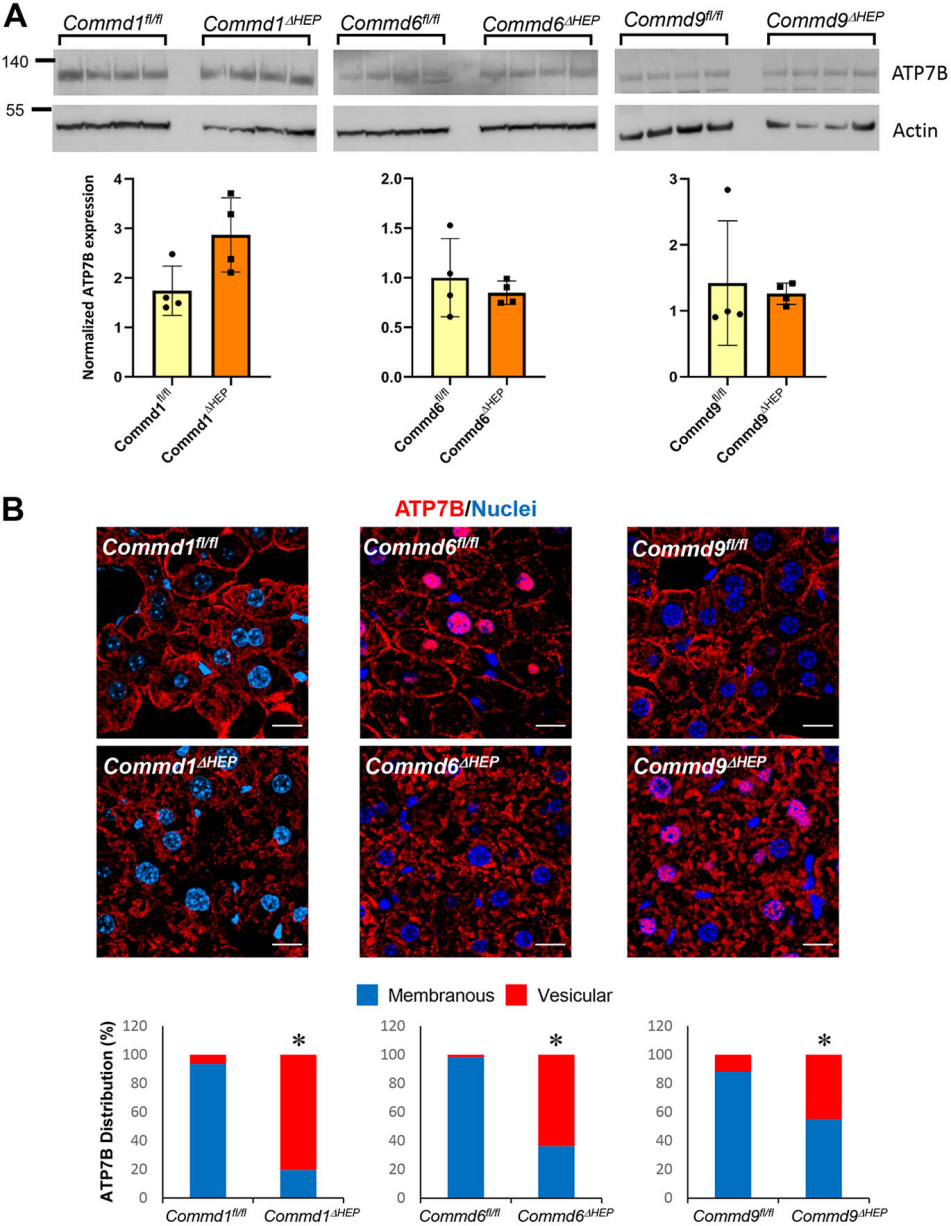
**ATP7B localization is altered in hepatic *Commd1*, *Commd6* or *Commd9* knockout mice.** (A) Immunoblot analysis for ATP7B expression in hepatocyte-specific *Commd1*, *Commd6* or *Commd9* knockout mice or corresponding floxed control animals (top). Quantification after normalization by the loading control (actin) (bottom). None of the knockout groups exhibited a statistically significant difference from the corresponding control (unpaired two-tailed Student's *t*-test). (B) Representative images of immunofluorescence staining for ATP7B (red) and nuclei (Hoechst, blue) in liver tissues of *Commd1^ΔHEP^*, *Commd6^ΔHEP^* or *Commd9^ΔHEP^* and their respective control (floxed) mice (top); *n*=4 for each genotype. Scale bars: 15 µm. Quantification of ATP7B distribution pattern in immunofluorescence staining of liver tissues (bottom). Four images were acquired from each mouse, and eight to ten cells were analyzed from each image (*n*=32-40 cells per mouse). *P<0.05 (Chi square test).

To assess the role of hepatic COMMD proteins in the regulation of copper homeostasis *in vivo*, we examined the ATP7B expression in liver tissue from hepatocyte-specific COMMD knockout mouse models. As shown in Fig. 3A, immunoblotting of the same membranes used in Fig. 1B revealed that COMMD protein deficiency does not result in altered expression of ATP7B in hepatocytes. However, immunofluorescence staining of the liver tissue showed that deficiency of COMMD1, COMMD6 or COMMD9 resulted in drastically altered ATP7B localization in hepatocytes (Fig. 3B). A substantial fraction of ATP7B localized to the plasma membrane of hepatocytes in wild-type mice, but liver tissue of *Commd1^ΔHEP^*, *Commd6^ΔHEP^* or *Commd9^ΔHEP^* mice displayed reduced plasma membrane staining and increased localization of ATP7B to cytosolic vesicular structures. These results indicate that COMMD protein deficiency leads to a defect in the transport of ATP7B from cytosolic vesicles to the plasma membrane.

### Intestinal deficiency of COMMD1 or COMMD9 does not impair copper handling

**Fig. 4.**
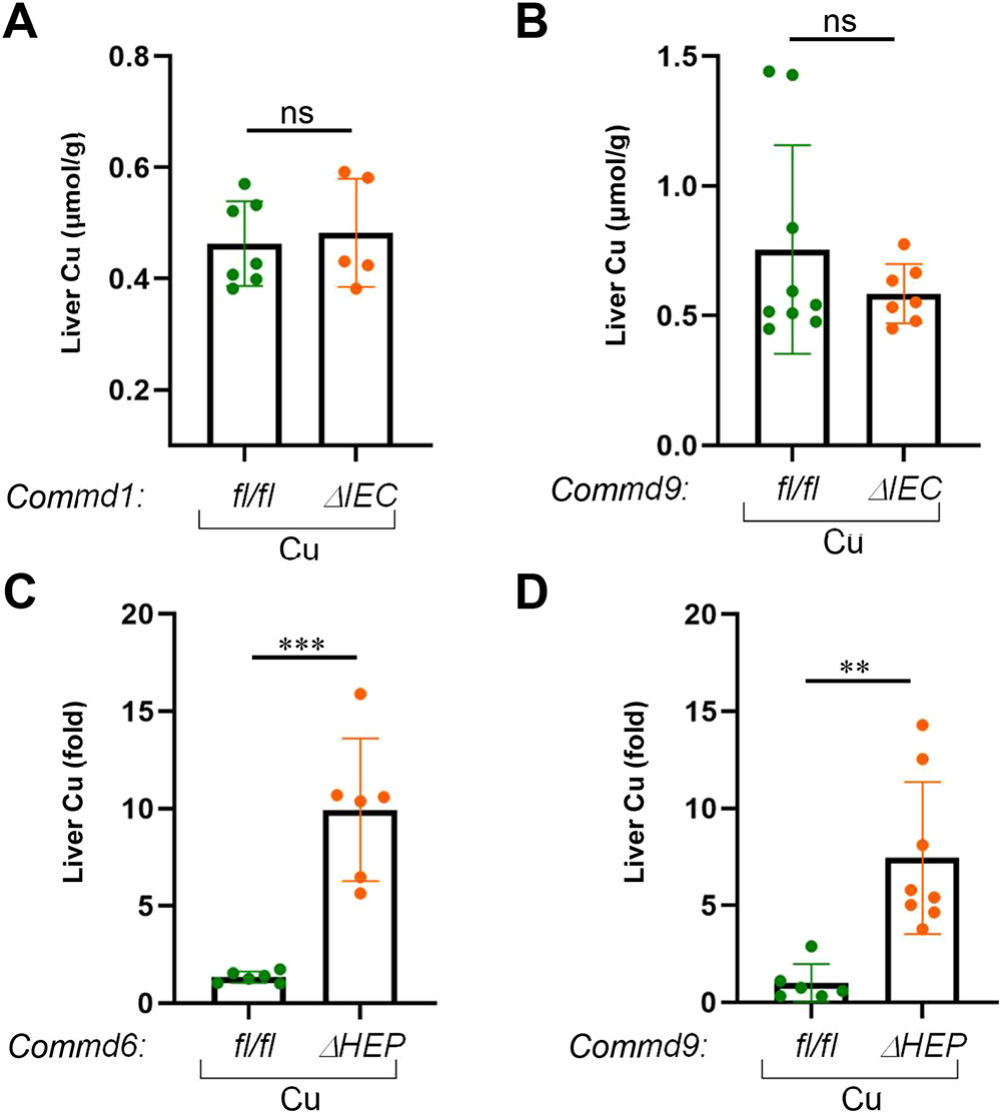
**Hepatic but not enteric knockout mice develop altered copper homeostasis.** (A) Hepatic copper contents (μmol/g) were measured in dried liver tissue of *Commd1^ΔIEC^* mice (*n*=7) and corresponding floxed control animals (*n*=5) on a high-copper diet. Results for individual mice are plotted along with the mean and s.e.m. for each group; ns, not significant (unpaired two-tailed Student's *t*-test). (B) Same analysis as in A, but for *Commd9^ΔIEC^* (*n*=9) and the corresponding littermate controls (*n*=7). (C) Hepatic copper concentrations were measured in dried liver tissue of *Commd6^ΔHEP^* (*n*=6) and corresponding floxed control animals (*n*=6) on a high-copper diet. (D) Same analysis as in C, but for *Commd9^ΔHEP^* (*n*=6) and the corresponding littermate controls (*n*=8). Data are presented as the fold change relative to the average value in the control floxed mouse group. **P<0.01; ***P<0.001 (unpaired two-tailed Student's *t*-test).

It is well known that hepatocyte COMMD1 deficiency results in accumulation of copper in the liver (Vonk et al., 2011); however, the contribution of this factor or the CCC complex to intestinal copper uptake has not been studied previously. To examine this, we first investigated the effect of intestinal COMMD protein deficiency on the amount of enterocyte and liver copper content in *Commd1^ΔIEC^* or *Commd9^ΔIEC^* mice. Initially, we challenged *Commd1^ΔIEC^*, *Commd9^ΔIEC^* or control mice with a copper-enriched diet by supplementing the drinking water with copper chloride (CuCl_2_) to a final concentration of 6 mM for 16 weeks (fed *ad libitum*; Fig. S2A). High dietary copper led to an increase in enterocyte copper levels compared with their respective water controls; however, deficiency of COMMD1 or COMMD9 did not alter the enterocyte copper levels (Fig. S2B,C). In contrast to enterocyte copper content, liver copper levels were unchanged after dietary copper treatment in control mice compared with water controls (Fig. S2D,E), indicating that dietary copper challenge is not sufficient to result in copper accumulation in the liver of normal mice. Likewise, the hepatic copper concentrations of *Commd1^ΔIEC^* or *Commd9^ΔIEC^* mice were unaffected by the copper-enriched diet when compared with control mice (Fig. 4A,B). Thus, in conditions of dietary copper excess, there was no overt phenotype when *Commd1* or *Commd9* was deleted from the intestinal epithelium.

Next, we challenged these mice with a copper-deprived diet by supplementing the drinking water with ammonium tetrathiomolybdate (TTM), a specific copper chelator that can be used to induce copper deficiency (Quinn et al., 2010). Mice were given TTM at a final concentration of 0.03 mg/ml in drinking water for 16 weeks (Fig. S2F). To assess the efficacy of TTM treatment to lower copper stores in mice, we examined serum ceruloplasmin levels in water- and TTM-treated mice. Ceruloplasmin is a copper-containing plasma protein and is reduced by copper deficiency (Olivares et al., 2008). As shown in Fig. S2G,H, non-mutant mice experienced a significant reduction in plasma ceruloplasmin levels in TTM-treated mice compared with their respective water controls. Importantly, ceruloplasmin levels were not substantially different in *Commd1^ΔIEC^* or *Commd9^ΔIEC^* mice compared with control animals, indicating that copper absorption was not impaired even in conditions of limited dietary supply. Altogether, these data demonstrate that ablation of COMMD proteins in intestinal epithelial cells does not result in appreciable effects on physiological copper balance.

### Liver deficiency of COMMD6 or COMMD9 results in abnormal copper accumulation

Previous studies demonstrated that liver-specific COMMD1-deficient mice are susceptible to hepatic copper accumulation (Vonk et al., 2011). In this regard, we examined whether hepatic ablation of *Commd6* or *Commd9* would mimic this phenotype. Again, we challenged *Commd6^ΔHEP^* or *Commd9^ΔHEP^* mice with excess dietary copper (added to drinking water) for 6 weeks and analyzed hepatic copper levels in these mice. As shown in Fig. 4C,D, *Commd6^ΔHEP^* or *Commd9^ΔHEP^* mice exhibited a significant accumulation of hepatic copper compared with the corresponding control animals. Altogether, our data suggest that liver-specific COMMD-deficient mice, but not intestinal-deficient mice, are susceptible to impaired copper homeostasis.

## DISCUSSION

COMMD proteins are highly conserved factors that have been linked to various physiological functions, including copper homeostasis (Phillips-Krawczak et al., 2015; van de Sluis et al., 2002; Vonk et al., 2011), inflammation (Li et al., 2014; Maine et al., 2007; Starokadomskyy et al., 2013), lipid metabolism (Bartuzi et al., 2016; Fedoseienko et al., 2018), adaptation to hypoxia (van de Sluis et al., 2010, 2007) and electrolyte transport (Biasio et al., 2004; Drévillon et al., 2011; Ware et al., 2018). Most of our knowledge is still centered on COMMD1, and comparatively much less is known about other COMMD family members. In fact, before this report, the functional role of other COMMD proteins in regulating copper balance *in vivo* was still unknown. Here, we report that, in addition to COMMD1, hepatic COMMD6 and COMMD9 also play a pivotal role in copper homeostasis. The studies presented indicate that hepatic deletion of *Commd1*, *Commd6* or *Commd9* results in loss of ATP7B at the hepatocyte plasma membrane, a finding that is correlated with elevated copper levels in all the models. Although cell-line studies show that COMMD1 deficiency leads to ATP7B accumulation in peripheral vesicles, this does not mean that these vesicles ultimately fuse with the plasma membrane to mediate copper efflux. One would have to surmise that plasma membrane fusion is indeed blocked, resulting in an inability of cells to excrete copper from lysosomes to the biliary canaliculus. In support of this, staining of liver sections shows that COMMD gene-deficient hepatocytes lose plasma membrane staining for ATP7B, suggesting that fusion events between lysosomes and the plasma membrane must indeed be blocked when CCC is defective.

The overlapping phenotype observed for hepatic deficiency of various COMMD genes was seen previously when studying plasma lipid homeostasis in these mice (Fedoseienko et al., 2018). Hepatic ablation of *Commd1*, *Commd6* or *Commd9* leads to elevated plasma LDL cholesterol levels owing to impaired endosomal trafficking of LDLR. The explanation for the phenotypic overlap between these models appears to be interdependence between these factors for protein stability (Fedoseienko et al., 2018; Phillips-Krawczak et al., 2015; Singla et al., 2019) and, at least at the hepatocyte level, deficiency of any of the three genes studied here seems to lead to a functional collapse of the CCC complex, with similar physiological consequences. Although these phenotypes are similar, suggesting that they hit on the same pathway, we also note that embryonic lethality is seen with each COMMD gene deletion in mice and, in each case, the embryonic phenotype is different. The reports in the literature that are most comprehensive pertain to *Commd1* and *Commd9* deficiency (Li et al., 2015; van de Sluis et al., 2007) and support the notion that these genes have unique developmental functions.

Previous studies demonstrated the involvement of the CCC complex in regulating copper-dependent ATP7A trafficking in cells (Phillips-Krawczak et al., 2015). In human cells, deficiency of the CCC complex resulted in accumulation of ATP7A in cytosolic vesicles and lack of dynamic movement of the transporter in response to changes in copper availability, much like the phenotype of ATP7B trafficking shown here in hepatocyte cell lines. However, until now, a potential role for COMMD proteins in the regulation of physiological activities of ATP7A *in vivo* has not been investigated. This question is important because whole-body deficiency of COMMD1 or other CCC complex components, as seen in humans with *CCDC22* and *VPS35L* gene mutations (Kato et al., 2020; Kolanczyk et al., 2015; Starokadomskyy et al., 2013; Voineagu et al., 2012), could potentially result in competing physiological effects in the liver, limiting copper excretion while also limiting copper absorption in the intestine. Here, we show that, at least at the level of the intestinal epithelium, we do not find any evidence of a physiological impairment of copper absorption in COMMD-deficient mice. Although our studies show minor changes in ATP7A in *Commd1^ΔIEC^* intestinal epithelium, we surmise that this change is not physiologically significant *in vivo* to result in an overt phenotype, because protein distribution remains normal in these enterocytes.

The CCC complex works as a crucial regulator of WASH, an actin nucleator that plays essential roles in endosomal organization and function (Alekhina et al., 2017). Furthermore, CCC and WASH work together with retromer and retriever, two evolutionarily related trimeric complexes involved in protein cargo selection in the endosomal compartment (McNally et al., 2017). Both retromer and retriever recognize cargo proteins either directly or indirectly by associating with cargo adaptors, such as the retromer-associated sorting nexin 3 (SNX3) or SNX27, and the retriever-associated SNX17 (McNally et al., 2017). Although ATP7A trafficking is known to be regulated by retromer/SNX27 in cell lines (Steinberg et al., 2013), any potential role for WASH, retromer or SNX27 in regulating ATP7A or ATP7B *in vivo* remains to be elucidated.

An interesting aspect of CCC deficiency models is the similarities and differences in the observed phenotypes in different species. As far as copper accumulation is concerned, COMMD1-deficient Bedlington terriers develop severe copper accumulation and pathological changes, ultimately resulting in cirrhosis of the liver (de Bie et al., 2006; van de Sluis et al., 2002). However, although hepatic *Commd1* knockout mice display an increase in hepatic copper, this does not result in liver histopathology (Vonk et al., 2011). This suggests that the hepatic copper levels in mice are not elevated to a degree that would result in hepatocellular toxicity, such as that as seen in Bedlington terriers with copper toxicosis. Indeed, Bedlington terriers with a null mutation in *COMMD1* accumulate very high levels of copper, which can exceed 10,000 mg Cu/g dry liver weight, similar to what is seen in humans with Wilson's disease (Kruitwagen and Penning, 2019). By contrast, mice develop much milder copper accumulation (never exceeding 400 mg Cu/g dry liver weight). Thus, the degree of copper accumulation is most directly correlated with liver injury and pathology. The reason for lower copper accumulation in mice is probably partial compensation of ATP7B trafficking in this species compared with dogs. Likewise, humans with *CCDC22* deficiency, which also impairs CCC complex function and trafficking of ATP7A in patient-derived fibroblasts, show no signs of liver injury or copper toxicosis (Phillips-Krawczak et al., 2015). These probands show only increased serum ceruloplasmin levels, a finding also observed in mutant Bedlington terriers and in *Commd1^ΔHEP^* mice, which reflects the mislocalization of ATP7B to cytosolic vesicles in the secretory pathway. By contrast, the alterations in plasma lipid homeostasis between these organisms are more highly concordant (Bartuzi et al., 2016; Fedoseienko et al., 2018).

We conclude from these studies that the impact of CCC complex dysfunction on various receptors and transporters is of variable significance in various tissues and organisms, depending on the specific target of interest. Moreover, the aggregate of these studies demonstrates that organismal models are essential in order to understand the physiological significance of impaired endosomal recycling resulting from CCC complex deficiency. Finally, given the redundant phenotypes of these models, a crucial question that remains unresolved is why all ten COMMD proteins are so highly conserved and what specific and non-redundant functions they support. At present, we lack a framework for the biochemical or structural features of these individual proteins to be able to answer these questions.

## MATERIALS AND METHODS

### Cell culture

Huh-7 cell lines were a gift from Dr Jay Horton [University of Texas (UT) Southwestern Medical Center, Dallas, TX, USA]. Huh-7 *CCDC93* knockout cells were generated using CRISPR/Cas9 editing technology and have been reported previously (Rimbert et al., 2020). All cell lines were cultured in high-glucose Dulbecco's modified Eagle's medium (DMEM) containing 10% fetal bovine serum and 1% penicillin/streptomycin, in incubators at 37°C, in air supplemented with 5% CO_2_. Cell lines were screened for mycoplasma, and all the experiments were performed on cells that tested negative. Cellular high-copper and low-copper treatments have been described previously (Phillips-Krawczak et al., 2015).

### Animal models

Mice were housed in barrier facilities and fed standard irradiated chow. All animal procedures were approved by the Institutional Animal Care and Use Committee (IACUC) and were under the supervision of the UT Southwestern Animal Resource Center (ARC) or were approved by the IACUC, University of Groningen (Groningen, The Netherlands). The conditional *Commd1* allele (*Commd1^fl/fl^*) and *Commd9* allele (*Commd9^fl/fl^*) have been described previously (Li et al., 2015; Vonk et al., 2011). *Commd1^fl/fl^* or *Commd9^fl/fl^* mice were bred with mice expressing Cre under the control of the *Villin* (also known as *Vil1*) gene to delete these genes in the intestinal compartment. Liver-specific *Commd1*, *Commd6* or *Commd9* knockout has been described previously (Fedoseienko et al., 2018; Vonk et al., 2011). ATP7A, ATP7B and CCC expression was performed in liver tissue and enterocytes isolated from 23- to 25-week-old mice of both sexes.

### Antibodies and chemicals

Antibodies to COMMD1 and COMMD9 were generated and reported previously (Li et al., 2015; Phillips-Krawczak et al., 2015). Antibodies to ATP7A and ATP7B were generated by Cocalico Biologicals (Reamstown, PA, USA), after immunization of rabbits with a peptide spanning amino acids 1406-1500 for ATP7A and amino acids 1372-1465 for ATP7B. The following antibodies were obtained from commercial sources: anti-VPS35L (Pierce, PA5-28553, 1:1000), anti-β-actin (Sigma-Aldrich, A5441, 1:5000), anti-GM130 (BD Transduction Laboratories, 610822, 1:200), anti-P84 (Genetex, GTX70220, 1:500), anti-CCDC22 (ProteinTech Group, 16636-1-AP, 1:1000) and anti-CCDC93 (ProteinTech Group, 20861-1-AP, 1:1000). Ammonium tetrathiomolybdate (TTM) and copper chloride II were purchased from Sigma-Aldrich.

### Enterocyte isolation

Mouse enterocytes were isolated from the middle section of the intestine (distal jejunum, proximal ileum). To eliminate any remaining luminal contents, the mid-intestine was flushed with Krebs-Ringer bicarbonate (KRB) buffer (10 mM D-glucose, 0.5 mM anhydrous MgCl_2_, 4.6 mM KCl, 120 mM NaCl, 0.7 mM anhydrous Na_2_HPO_4_, 1.5 mM anhydrous NaH_2_PO_4_ and 15 mM NaHCO_3_, pH 7.4) supplemented with 1 mM dithiothreitol, followed by occlusion of one end with a suture. The intestinal tube was filled with KRB buffer containing 10 mM EDTA and 1 mM dithiothreitol, and the other end was closed with a suture. The sealed intestine was placed in a 50 ml conical tube containing KRB buffer, followed by incubation at 37°C with agitation for 20 min. After 20 min, the sealed intestine was gently palpated to loosen and dissociate intestinal crypts from the mucosa. One end was cut open, and the contents were drained into a microfuge tube. Cells were collected by centrifugation at 300 ***g*** for 5 min, and the resulting cell pellets were rinsed gently in KRB buffer three times.

### Protein extracts and immunoblotting

Enterocyte and liver tissue lysates were prepared by adding tissue lysis buffer (50 mM Tris-HCl, pH 7.5, 250 mM NaCl, 3 mM EDTA, 3 mM EGTA, 1% Triton-X 100, 0.5% NP-40 and 10% glycerol) supplemented with protease inhibitors (Roche). Immunoblotting experiments were performed by resolving the protein samples using 4-12% gradient Novex Bis-Tris gels (Invitrogen) and transferring them to nitrocellulose membranes. Multiple proteins with different molecular weights were detected on the same membrane by cutting the membranes that resulted in the same control blots for Fig. 1A,B, Fig. 2A and Fig. 3A. Membranes were blocked with 5% milk solution in Tris-buffered saline (50 mM Tris, pH 7.4, 150 mM NaCl) containing 0.05% Tween-20, followed by incubation with primary antibodies and then with horseradish peroxidase-conjugated secondary antibodies. The chemiluminescent blots were imaged using a ChemiDoc imager (Bio-Rad), and the densitometric analysis was performed using ImageJ software.

### Immunofluorescence

Immunofluorescence staining of cells was performed as previously reported. Intestinal tissues were fixed overnight at 4°C in PBS containing 4% paraformaldehyde (PFA); thereafter, the tissue was washed three times with PBS and placed in 70% ethanol. Tissue processing (paraffin embedding and sectioning) was performed by the UT Southwestern Molecular Pathology Core. Paraffin-embedded slides were deparaffinized, and antigen retrieval was performed by incubation in citrate buffer (Sigma-Aldrich). Liver tissues were fixed at room temperature for 2 h using a fixative solution containing 2% PFA, 0.1 M lysine, 0.01 M sodium (meta)periodate in sodium phosphate buffer (0.1 M Na_2_HPO_4_ and 0.1 M NaH_2_PO_4_, pH 7.4). Thereafter, the tissue was rinsed and kept in 0.5 M sucrose solution in sodium phosphate buffer overnight at 4°C. Samples were then removed and embedded in cryomolds with OCT compound, followed by cryosectioning that was performed by the UT Southwestern Molecular Pathology Core. After cryosectioning, the slides were washed with PBS.

All the slides (intestinal and liver sections) were incubated in blocking buffer (PBS supplemented with 3.5% normal goat serum) in a humidified chamber, followed by incubation with primary antibodies overnight at 4°C. Thereafter, the samples were rinsed in PBS and then incubated with secondary antibodies for 1 h at room temperature. Alexa Fluor 555 goat anti-rabbit or Alexa Fluor 488 goat anti-mouse were used as secondary antibodies. Nuclear stain (Hoechst 33342) was added, and the coverslips were mounted using SlowFade Gold Antifade reagent (Life Technologies, Grand Island, NY, USA).

### Image quantification

Images were obtained using an A1R confocal microscope (Nikon) equipped with the NIS-Elements AR (Nikon) software. Acquisition settings for imaging were identical for all cell and mouse genotypes. Images were analyzed using FUJI ImageJ (https://imagej.nih.gov/ij/). Quantification of ATP7A in intestinal epithelial cells was performed by defining three regions of interest (ROIs) in each image. Each ROI was drawn manually to include between eight and 12 enterocytes, and this was performed by a team member who did not acquire the images and who was blinded to the genotype of the animals. Two to four independent images per animal were processed. On these images, we measured the mean fluorescence intensity for ATP7A (red channel) and for the nuclear Hoechst 33342 stain (blue channel). ATP7A fluorescence intensity was then normalized to nuclear fluorescence intensity (as a measure of the number of cells in each ROI). Quantification of the ATP7B staining pattern in liver sections and Huh-7 cells was performed by marking individual cells for qualitative pattern rating. This was performed by a team member who did not acquire the images and who was blinded to the identity of the samples. Data were then processed with Excel (Microsoft) and plotted with Excel or Prism v.6 (GraphPad).

### Measurement of copper levels

Copper levels were determined in a routine diagnostic laboratory. Ten milligrams of mouse liver or enterocyte homogenate were dried overnight at 105-110°C, and the weight of the dried tissue was determined. Subsequently, the dried tissue was incubated in 500 µl of lysis solution containing HClO_4_ and HNO_3_ (4:1) for 3 h at 60°C. At room temperature, 4500 µl of H_2_O was added to the lysed tissue, and 100 µl was diluted further in a solution containing 1% HNO_3_ and 0.01% Triton-X 100, and the diluted sample was measured in an inductively coupled mass spectrometer (ICP-MS). A copper reference was included to calculate the copper concentration in the tissue.

### Plasma ceruloplasmin assay

Plasma ceruloplasmin levels were determined using a ceruloplasmin colorimetric activity kit (Thermo Fisher Scientific) according to the manufacturer's instructions.

## Supplementary Material

Supplementary information
