# Supplementary material for: Regulation of murine copper homeostasis by members of the COMMD protein family
Source: Dis Model Mech. 2021 Jan 8;14(1):dmm045963. doi: 10.1242/dmm.045963 (PMC7803461; doi:10.1242/dmm.045963)
Supplement: Supplementary information [file dmm-14-045963-s1.pdf]

Figure S1

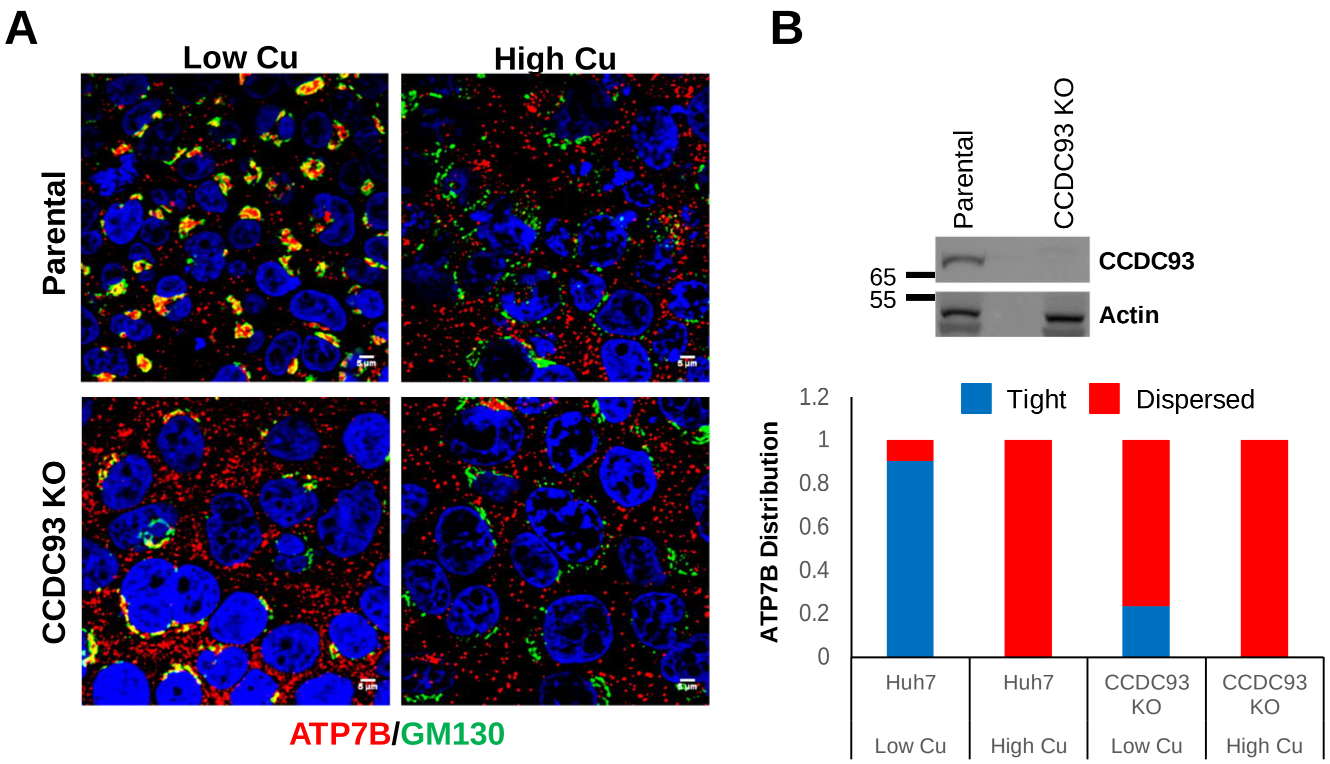

Figure S1: Depletion of CCDC93 impairs ATP7B trafficking. (A) Huh-7 parental cells and a CCDC93 knockout sub-line were exposed to high or low copper-containing media. Following this, cells were immunostained for ATP7B (red) and the Golgi marker GM130 (green). A nuclear dye was also used (blue). Immunofluorescence images are shown. Scale bar, 5µm. **(B)** Immunoblot for CCDC93 and actin (top panel) and quantification of ATP7B distribution pattern in the indicated cell lines.

Disease Models & Mechanisms • Supplementary information

Figure S2

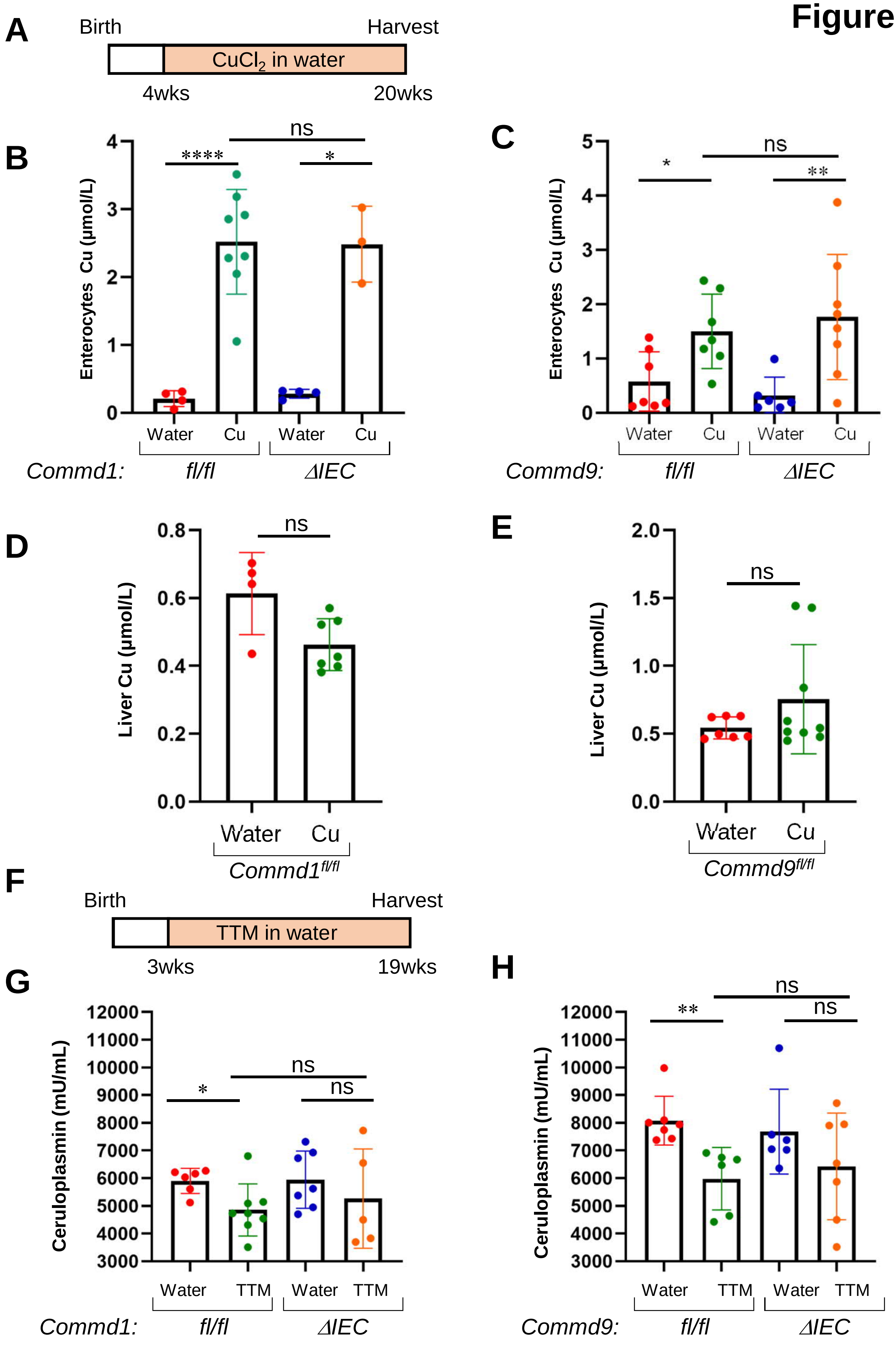

Disease Models & Mechanisms • Supplementary information

Figure S2: Enteric *Commd* knockout does not impair copper balance. (A) Schematic depicting copper treatment course. **(B)** Enterocyte copper concentrations (μmol/L) were measured in isolated enterocytes of *Commd1*$^{\Delta IEC}$ mice (water control: n=4, copper treated: n=3) and corresponding floxed control animals (water control: n=4, copper treated: n=8) at the conclusion of the high copper diet treatment depicted in (A). Results for individual mice are plotted along with the mean and s.e.m. for each group; $*P < 0.05$, $****P < 0.0001$ and ns, not significant (unpaired two-tailed t test). **(C)** Same analysis as in (B) but for *Commd9*$^{\Delta IEC}$ (water control: n=6, copper treated: n=8) the corresponding littermate controls (water control: n=7, copper treated: n=7). $*P < 0.05$, $**P < 0.01$ and ns, not significant (unpaired two-tailed t test) **(D)** Hepatic copper concentrations were measured in dried liver tissue of *Commd1*$^{fl/fl}$ animals on a water (n=4) or high copper diet (n=7). Results for individual mice are plotted along with the mean and s.e.m. for each group; ns, not significant (unpaired two-tailed t test). **(E)** Same analysis an in (D) but for *Commd9*$^{fl/fl}$ animals on a water (n=7) or high copper diet (n=9). **(F)** Schematic depicting tetrathiomolybdate (TTM) treatment course. **(G)** Ceruloplasmin levels (mU/mL) were measured in plasma of *Commd1*$^{\Delta IEC}$ mice (water control: n=7, TTM treated: n=5) and corresponding floxed control animals (water control: n=6, TTM treated: n=8) on TTM or control (water) diets. Results for individual mice are plotted along with the mean and s.e.m. for each group; $*P < 0.05$, and ns, not significant (unpaired two-tailed t test). **(H)** Same analysis as in (G) but for *Commd9*$^{\Delta IEC}$ mice (water control: n=6, TTM treated: n=7) and corresponding floxed animals (water control: n=7, TTM treated: n=6). $**P < 0.01$ and ns, not significant (unpaired two-tailed t test).

Disease Models & Mechanisms • Supplementary information
